# Systematic Review and Meta-Analysis of Thermal Stress Assessment in Poultry Using Infrared Thermography in Specific Body Areas

**DOI:** 10.3390/ani14223171

**Published:** 2024-11-06

**Authors:** Roberto Carlos Hernández-Sánchez, Francisco Ernesto Martínez-Castañeda, Daniel Alonso Domínguez-Olvera, Maria Elena Trujillo-Ortega, Víctor Manuel Díaz-Sánchez, Ezequiel Sánchez-Ramírez, Elizabeth Posadas-Hernández, Itzayana Mejía-Flores, Elein Hernandez

**Affiliations:** 1 Facultad de Estudios Superiores Cuautitlán, Universidad Nacional Autónoma de México, Km 2.5 Carretera Cuautitlán-Teoloyuca, Cuautitlán Izcalli C.P. 54714, Mexico; rh00760@gmail.com (R.C.H.-S.); victordiaz@cuautitlan.unam.mx (V.M.D.-S.); nayazait@gmail.com (I.M.-F.); 2 Instituto de Ciencias Agropecuarias y Rurales (ICAR), Universidad Autónoma del Estado de Mexico, Toluca C.P. 50295, Mexico; femartinezc@uaemex.mx; 3 Facultad de Medicina Veterinaria y Zootecnia, Universidad Nacional Autónoma de México, Av. Universidad #3000, Coyoacán, Mexico City C.P. 04510, Mexico; iazdalonso@gmail.com (D.A.D.-O.); elenam@unam.mx (M.E.T.-O.); ezequiel.sanchez02@gmail.com (E.S.-R.); eposadas63@yahoo.com.mx (E.P.-H.)

**Keywords:** animal welfare, cold stress, heat stress, infrared thermography, meta-analysis, poultry, thermal stress, thermography

## Abstract

Thermal stress is a health and welfare concern in the poultry industry. Poultry have specific thermoregulation strategies for heat stress (i.e., vasodilatation) or cold stress (i.e., vasoconstriction). Infrared thermal (IRT) analysis is a non-invasive temperature assessment technology with significant benefits compared to conventional temperature measurements, which are invasive and time-consuming. However, a wide range of IRT methodologies and equipment are used for temperature assessment in poultry. The aim of this study was to perform a systematic review and meta-analysis of IRT applications in poultry undergoing thermal stress. The bibliographic search yielded 17 records for qualitative synthesis and 10 for quantitative analysis. The results showed IRT is more commonly studied during heat stress than cold stress, and more research is being conducted on laying hens than other poultry species. Also, four body areas (parts of the head, body, face, and leg) were identified as common areas of interest for body surface temperature measurement. There is a clear thermoregulation response to thermal stress in poultry, with marked differences between featherless and feather-covered areas. IRT in poultry undergoing thermal stress has a good diagnostic value and represents an important welfare assessment tool for future research, particularly when combined with other welfare assessment methods.

## Simple Summary

Thermal stress is a significant issue in the poultry industry. It is associated with health and welfare concerns, physiological and behavioral responses, and production and economic losses. Thermal stress includes both heat and cold stress and affects all poultry species. Core body temperature changes during thermal stress can be assessed with conventional methods, such as a cloacal thermometer. However, this method is invasive and time-consuming, while infrared thermography (IRT) is a non-invasive method capable of capturing the thermal radiation emitted by the skin’s surface. This work aimed to perform a systematic review and meta-analysis concerning different IRT applications in poultry undergoing thermal stress. Overall, four body areas (parts of the head, body, face, and leg) were identified as common areas of interest for body surface temperature measurement with IRT in laying hens, broilers, and turkeys. Featherless body areas were valuable for thermoregulation. However, the findings demonstrated a degree of thermal response that depends on the age, species, thermal treatment, and body area.

## 1. Introduction

The poultry meat and egg industries are expected to continue growing worldwide. In 2032, the poultry meat industry is expected to represent 41% of the consumed meat sources; similarly, egg consumption (kg/cap) is expected to increase from 10.9 to 11.6 between 2019 and 2031, respectively [1]. However, several environmental stressors, such as heat and cold stress, affect meat and egg production and present poultry welfare challenges.

Poultry are homeothermic animals with a body temperature of approximately 40–42 °C [2]. The optimum temperature for performance (thermal comfort zone) is 19–22 °C, and thermal stress signs start to show when the environmental temperature rises above 25 °C (heat stress) or decreases below 16 °C (cold stress) [3,4,5]. Environmental temperature and relative humidity are the main climatic factors that affect poultry performance [6], but other animal-based factors, including the phenotype and anatomic–physiological qualities, could hamper thermoregulation efficiency [7].

Poultry have unique physiological and anatomical traits (e.g., high metabolic activity, plumage, lack of sweat glands, etc.) that may hamper their thermoregulation response if there is an imbalance between the amount of heat energy produced by the animal and the surrounding environmental temperature [8]. The thermoregulation response will depend on the type of stressor. Under heat stress, poultry attempt to dissipate heat through cutaneous and evaporative loss via the respiratory route with panting [3,8]. This response is also accompanied by other physiological (e.g., increase in plasma cortisol, changes in heterophil-to-lymphocyte ratio) and behavioral responses (e.g., panting, changes in feed and water intake and drinking) that eventually affect the animals’ welfare and egg and meat quality [3,4]. Under cold stress, there is a vasoconstriction response to reduce heat loss on the body’s surface, accompanied by other physiological and behavioral responses that may include gluconeogenesis and lipolysis to increase glucose accessibility and increased feed intake to sustain the energy demand [5,9]. These thermoregulation strategies occur across the bird’s body surface and core body temperature but may be affected by the presence or absence of feathers. Feathers have thermal insulation properties that prevent heat loss during cold stress but may hinder heat dissipation during heat stress [8]. In addition, there are superficial temperature differences between feather-covered vs. featherless areas. Featherless areas such as the leg, comb, wattle, earlobe, and eye have a higher surface temperature than feather-covered areas (wing, back, chest) during thermal comfort and heat stress [2]. These featherless body areas are also called “biological, thermal windows” and have poor insulation and, subsequently, higher vasoconstriction/vasodilation capacity [10]. However, there is no standard image recording protocol for birds’ body parts, and multiple body regions have been reported with no clear inclusion criteria. For example, the mean head/face surface is a commonly examined area, but studies often also include measurements of specific parts of the head like the beak, comb, eye, wattles, and earlobe.

The conventional method for body temperature assessment in poultry is cloacal thermometry, but it is considered invasive, time-consuming, and likely stressful for the animal [2,7]. Infrared thermal (IRT) imaging is a noninvasive technology based on infrared radiant emission from the bird’s surface. Several studies have detailed the effect of experimentally induced thermal stress on poultry body parts and described specific age and management practices [3,6,7,9,11,12,13,14]. However, a quantitative meta-analysis on thermal stress’s effect on poultry body area temperature, species, and age has not been conducted. A meta-analysis can objectively interpret contradictory results in the literature and overcome small sample size limitations [15].

The objective of the present study was to synthesize and critically analyze the results of different empirical studies on thermal stress on specific body areas and the effect on age and species in laying hens, broiler chickens, and turkeys.

## 2. Materials and Methods

The development of the present work included a literature review of publications related to applying thermographic analysis to thermal stress (i.e., heat stress and cold stress) in poultry research. The methodological procedure included an information search, systematic review, quantitative analysis, analysis of categories, and statistical analysis. Bioethics committee approval was not required for this study because no animals were used.

This systematic review and meta-analysis were performed according to the Preferred Reporting Items for Systematic Reviews and Meta-analyses (PRISMA) recommendations [16]. Relevant studies concerning infrared thermography applications for poultry flock health monitoring during thermal stress in laying hens, broiler chickens, and turkeys were selected and assessed.

### 2.1. Information Sources

**Figure 1:**
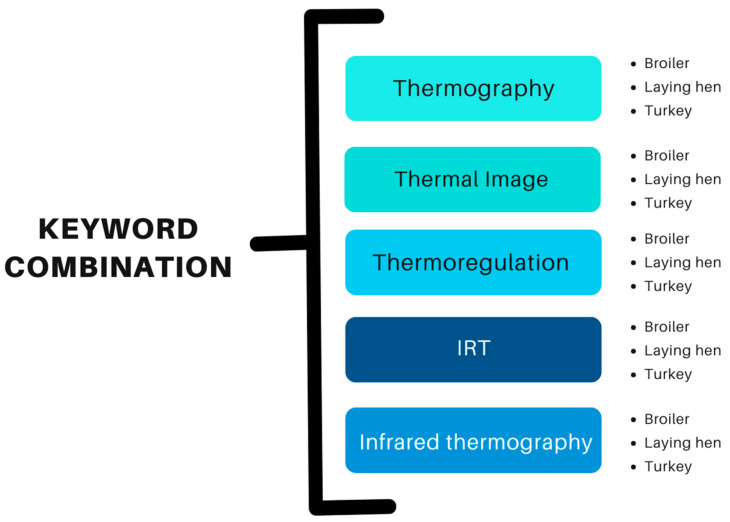
Keyword combination for article search.

The bibliographic search used Google Scholar and Science Direct as reference sources. The search included articles published between 1 January 2000 and 10 November 2023. The keywords and keyword combinations used are described in Figure 1. The following key terms for the literature search were used: thermography, thermal image, thermoregulation, IRT, infrared thermography, laying hen, chicken, broiler chicken, and turkey.

### 2.2. Selection Criteria

**Figure 2:**
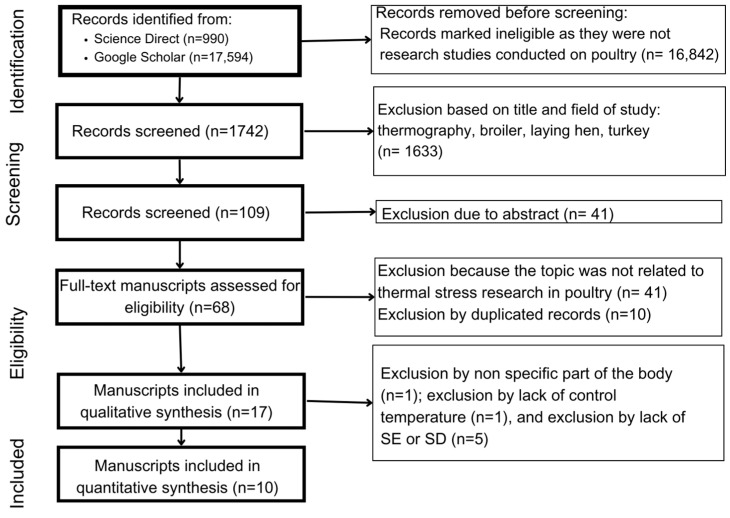
Literature funnel (PRISMA diagram).

The selection process is graphically illustrated in Figure 2. Two reviewers (EH and RH) performed the review based on the selection and exclusion requirements below:

1. The studies had to include laying hens, broiler chickens, and turkeys as study subjects;
2. The studies had to contain a thermoneutral group and at least one group experiencing thermal stress (i.e., control group compared to heat or cold stress-affected group);
3. The studies had to include a body temperature control to confirm the accuracy of thermographic changes;
4. The studies had to contain results in at least two parts: the start and endpoints of thermal stress;
5. The studies had to contain results of thermographic changes in different parts of the body;
6. The studies had to include results presented as means with standard deviations (SDs) and/or standard errors (SEs). Articles that reported nonparametric results and results including ranges or subjective values were excluded.
7. The papers had to be written in English.
8. Reviews and opinion papers were excluded.

### 2.3. Data Analysis

All data processing and analysis were performed using RStudio for R software V 4.3.2 (Vienna, Austria: R Foundation for Statistical Computing). The effect size (control -treatment) and standard error of the effect size were analyzed with the “esc” package (Version 0.5.1). The assessment of the effect size in conjunction with a Hartung–Knapp random effects adjustment, confidence interval, estimation hypothesis testing, heterogeneity test, and heterogeneity coefficient were performed with the “meta” package (Version 7.0-0). The evaluation of the fixed effect was performed with the “dmetatools” package (Version 1.1.1), and the development of the forest graph was performed with the “tidyverse” package (Version 2.0.0). Dispersion averages were estimated based on the mean, standard deviation, and number of observations for each indicator under study. A random-effects model was utilized to test the hypothesis of heterogeneity, the average standard difference in the effect, and its confidence interval (α = 0.05); chi-square tests supported this decision [17]. Differences were considered significant at *p* < 0.05.

## 3. Results

A total of 18,584 articles were initially selected, but only 1742 were selected based on the research criterion; 16,842 publications were excluded because they were not conducted on poultry and because keyword combinations included homonyms (e.g., turkey for the animal, Turkey for the country) (Figure 2). Based on title and abstract screening (for mentions of IRT application in poultry), we further excluded 1633 and 41 publications, respectively, that did not mention the IRT applications in poultry (Figure 2). Of the remaining 68 publications, 41 were excluded due to not including either heat or cold stress assessment, and 10 duplicates were removed. The information analysis was divided into two stages: qualitative and quantitative syntheses (Figure 2).

### 3.1. Categories of Analysis

The qualitative synthesis included 17 records classified by poultry species: laying hens, broiler chickens, and turkeys. A qualitative summary of the main findings was presented without attempting to quantify the effect sizes due to high between-study variation in result outcomes and methodologies.

The quantitative analysis was performed for 10 of the 17 publications since not all records included all the criteria for the meta-analysis process and were classified by thermal stress (Table 1). The studies were characterized based on the nature and type of thermal stress (i.e., cold or heat stress) and species or farm production type (laying hen, broiler chicken, and turkey) (Table 1). Additionally, thermographic areas of interest on the bodies of the birds were identified and classified for analysis (Table 2). Due to different thermal sites of interest across studies, the body areas were grouped into four areas (head, face, body, and leg) for further analysis (Table 2). The head part classification comprised records that included the beak, comb, eye, earlobe, or wattles. However, some records made specific distinctions between specific head parts and an overall head or face area analysis; in addition, some compared the left and right sides of the face. Therefore, the general head or face analysis was classified as an independent body area if the records made the above distinctions. The body area consisted of measures of either the chest, back, wing, or overall body. The chest area was not included in the cold stress meta-analysis because only one study measured it [11]. The leg area included records that described either the shank or leg.

### 3.2. Qualitative Synthesis

The topic with the highest number of publications on the application of thermal imaging in poultry under thermal stress is represented by heat stress across poultry species, while laying hens are the most commonly studied poultry type in thermal stress studies. Brazil (47%) and the USA (23%) have the most publications on thermal stress. The included articles cover various topics, including animal characteristics [2,3,6,7,13,14,25], climatic factors [9,11,22,26,27,28,29,30], and management practices [12,31] associated with thermal stress measured using IRT.

Several IRT methodologies are used in the reviewed records, including the distance between the camera and the animal, selected thermal emissivity parameters for featherless and feather-covered areas, and camera models that inherently involve different technical features such as the measurement uncertainty degree, noise equivalent differential temperature (NEDT), and focal plane array (FPA) (Table 1).

#### 3.2.1. Thermal Stress Assessment in Laying Hens Using IRT

All of the studies reviewed in this section were conducted in controlled experimental facilities; the hens were housed in cages, usually individually [26] or in small groups of 1 to 3 birds per cage [3,7,12,25,31]. Two studies focused on hens in group pens with different group sizes: 8 [26] and 200 [13] hens per group. Birds were of various ages and strains, but most studies included adult laying hens, while two included chicks and pullets [2,22]. Nonetheless, the surface temperature measures were performed individually. Thermal imaging acquisition was conducted with different equipment types, and the experimental setup was not evident in all experiments (Table 1). Most of the studies performed thermal imaging at a horizontal distance of about 0.8 to 1.5 m away from the birds, and the camera parameters were set to 0.98 thermal emissivity [2,3,12,13,22,26,31]. Loyau and colleagues determined different thermal emissivity parameters for featherless (0.98) and feather-covered (0.896) areas [13].

Overall, the reviewed articles described poorer welfare outcomes for birds experiencing thermal stress treatments than the control groups. Some took repeated measures over time, demonstrating how the birds’ welfare level changed days [3] or weeks [2,13,22] after the initial thermal stressor. In addition to surface temperatures, other animal-based welfare indicators that were addressed include heterophil/lymphocyte ratio [3], corticosterone blood level [3], feather quality [13], respiratory rate [25], and egg quality [13]. The main resource-based indicators were air temperature, relative humidity, and air motion.

Heat stress was the most assessed thermal event and was evaluated using different level exposures (mild vs. moderate vs. severe heat stress). Each study allocated the temperature range differently based on its research objectives. Nonetheless, a universal finding was that the welfare level decreased as the severity of the exposure increased. For example, laying hens exposed to high temperatures in thermal environment-controlled settings have higher rectal and body surface temperatures compared to medium-heat- and low-heat-exposed hens. These findings are aligned with other physiological and behavioral responses [3,25]. Similar findings in non-controlled environments were reported by Souza et al. [26], who assessed a laying hen lineage (*Naked Neck*) in two climate types (semi-arid and tropical). The laying hens in question in the tropical area presented higher body surface temperatures. In addition, there were differences between feather-covered and featherless areas.

Most studies described the roles of feathers and of the body areas assessed by thermal imaging; a detailed quantitative analysis can be found in Section 3.3. The most evaluated body areas across the studies were the head, neck, chest, back, wings, and legs. Kim and colleagues [7] identified five distinct regions of the head (eyes, earlobes, wattles, comb, beak, and nose) for thermal analysis at different time points in the day in hens undergoing cold and heat stress (10 ± 4 °C and 30 ± 2 °C, respectively) compared to hens housed at optimal ambient temperature (22 ± 2 °C). The authors reported the highest maximum surface facial temperature at 12:00 h in heat-stressed hens, at 45.41 ± 0.96 °C, and the wattle and comb were the facial regions contributing in high proportion (62.2% and 18.3%, respectively) to the overall head temperature in heat-stressed hens. Similar findings were described in laying pullets of 17 weeks of age undergoing mild and moderate heat stress (ambient temperature: 25 °C and 30 °C, respectively) with high surface temperature values in the comb, followed by eye and head temperatures. In the study, other head appendages were not considered [2]. Mean head surface temperature values are described in other studies but with no specific facial areas of interest [3,22]. The leg is another featherless area in the laying hen with higher thermal surface temperatures when ambient temperatures are above thermoneutral zones [2,3,13,22,26]. Souza et al. reported the highest values of surface temperatures in the neck, followed by the face and legs, but this was due to the hen lineage being characterized by a featherless neck, which allows for heat dissipation. Conversely, feather-covered areas of the body, such as the chest, wings, and back, had a lower surface temperature increase and tended to be closer to the air temperature [2,3,13,26].

Thermal mapping was also valuable in identifying thermal changes associated with cold stress. Kim et al. [7] identified significant temperature changes compared to heat stress and hens in control group. The lowest facial temperature was recorded at 5:00 h at the beak region (23.22 °C) and the maximum facial surface temperature at 12:00 (41.01 ± 0.96 °C). The areas around the eyes and wattles comprised 77.8% of the maximum facial surface temperature values. Similarly, Andrade et al. [22] described a decrease in head temperature as the air temperature decreased in mild- and moderate-cold-exposed birds. In addition, the mean surface temperatures with the lowest values were body surface temperature, followed by shank and head temperature in pullets.

Some studies employed IRT to assess specific animal and management conditions and their welfare outcomes. Souza et al. [26] studied a genetic trait that provides a phenotype with a featherless neck, resulting in the highest mean body temperature compared to other body areas, as described above. Meanwhile, Ramamneh et al. [12] assessed the effects of partial comb and wattle removal at prolonged high environmental temperatures. The study confirmed these body appendages were critical for thermoregulation during high temperatures. Mortality and behavioral changes were observed more than in the control groups (untrimmed hens). The body temperature was higher than that of control hens at 48 h from the beginning of the heating episode in hens 191 days of age. Other alternative management applications were explored by researchers trying to address the welfare issues associated with heat stress. Chepete and colleagues [31] studied the efficacy of partial body surface cooling by intermittent sprinkling. Their findings included reduced mortality (40% vs. 100%) and body temperature increase (2.2 ± 0.3 °C vs. 2.9 ± 0.1°C) compared to the control group with no sprinklers. Acclimatization during the early stages has been suggested to aid in coping with heat stress. Candido et al. [2] did not identify an acclimatization effect between different rearing conditions for cloacal and body part surface temperatures. Nonetheless, their study reported a positive linear relationship between surface temperatures and cloacal temperature, highlighting thermal imaging as an alternative to direct cloacal temperature measurement.

#### 3.2.2. Thermal Stress Assessment in Broilers and Turkeys Using IRT

Most of the studies reviewed in this section were conducted in controlled experimental facilities; only three were conducted in broiler chicken farms [6,27,29]. Similar strains and age groups were assessed across all the studies. Most broiler chicken studies reported using COBB flocks [6,9,14,27], and two age groups were identified, including birds between 1 and 35 days of age [6,14,29] and from 36 to 61 days of age in broiler chickens [27,28]. Age differences were also considered in the two studies that included turkeys [11,30].

Different IRT technologies and methodologies were employed; the most common infrared cameras were FLIR Systems [6,11,28,30] and Testo [9,27,29] (Table 1). A few studies described the experimental setup, which included thermal emissivity of 0.94 and 0.95 and the distance between the camera and the animal [9,27,30]. Thermal imaging in broilers mainly included mean surface temperature assessment [9,14,27,28,29] and only one study aimed to evaluate body temperature compared to facial surface temperature [6]. Conversely, other body areas like the head, chest, wing, and legs were included in the thermal imaging of turkeys [11,30].

Overall, the studies revised in this section are grouped as either studies assessing thermal stress (see Table 1) or studies assessing the practices of climatic factor management and their effect on body surface temperature [27,28,29,30]. Similar to thermal imaging in laying hens, the most studied thermal stress in broiler chickens was heat stress, resulting in increased surface temperatures. Giloh et al. [6] reported a positive correlation between body core and facial temperature. Similarly, Nascimento et al. [9] described increased mean surface temperatures in birds housed at 32 °C air temperature compared with 25 °C and 18 °C air temperatures. However, the authors described no age effect on the mean surface temperature. The lowest mean surface temperature was identified in 35-day-old birds and was associated with thick feather coverage at that age. In contrast, Fantin et al. [14] analyzed the surface temperatures and heat dissipation of heat under cold stress. The authors confirmed greater dissipation when the birds are housed in temperatures below their thermal comfort zone. Likewise, Mayes et al. [11] reported decreases in feather-covered surface temperatures in turkeys reared at 4 °C below the recommended rearing temperatures compared to control turkeys from weeks 1 to 12 of age. The authors also generally identified no significant differences in surface temperatures in featherless areas (head and legs) compared to the control groups, with a few exceptions on specific time points.

The other cohort of studies assessed cooling technologies, including fans, fogging systems, and cooling pads. These studies used IRT to describe changes in mean body surface associated with air velocity, air temperature, heat index, and broiler chickens’ age [27,28,29]. Uemura et al. [30] reported similar findings, where higher air ventilation rates reduce the air temperature and hence the heat stress in 20-week tom turkeys. The authors describe a significantly higher mean surface body temperature (31.6 ± 0.9 °C) compared to three ventilation rates (VR-50: 29.9 ± 1.1 °C; VR-75: 29.0 ± 1.0 °C; VR-100: 29.7 ± 1.3 °C) with a similar surface temperature change in the head, torso, and legs of turkeys.

### 3.3. Quantitative Synthesis

Ten studies were fully reviewed and selected for meta-analysis based on the research objective (Table 1). Eight studies were included due to containing thermal change analyses that compared heat stress and control groups across ages and species, while five studies were assessed for cold thermal changes. Some studies discussed both cold and heat stress-exposed groups; the information was extracted accordingly.

#### 3.3.1. Analysis of Studies That Investigated Body Temperature Changes in Poultry During Heat Stress 

**Figure 3:**
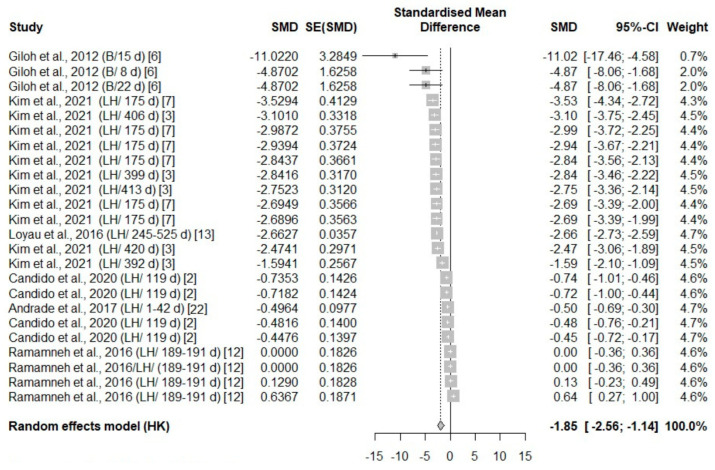
Mean difference in the parts of the head surface temperature of broiler chickens (B) and laying hens (LH) of different days of age (d) under heat stress. Effect size (SMD, Standard medium difference); SE (standard deviation of SMD) [2,3,6,7,12,13,22].

**Figure 4:**
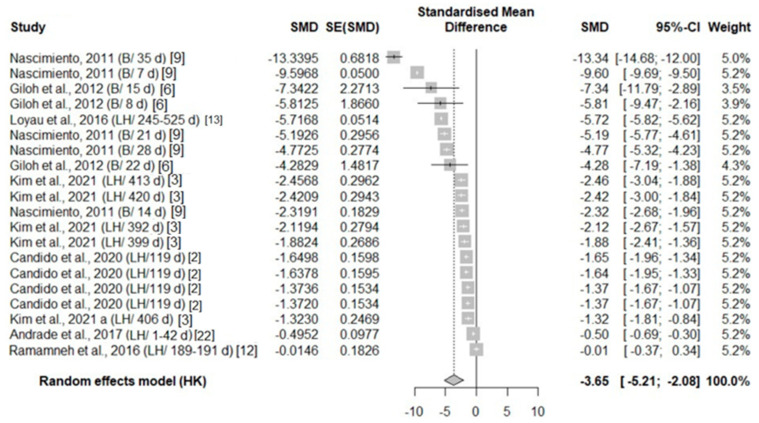
Mean difference in body surface temperature of broiler chickens (B) and laying hens (LH) of different days of age (d) under heat stress. Effect size (SMD, Standard medium difference); SE (standard deviation of SMD) [2,3,6,9,12,13,22].

**Figure 5:**
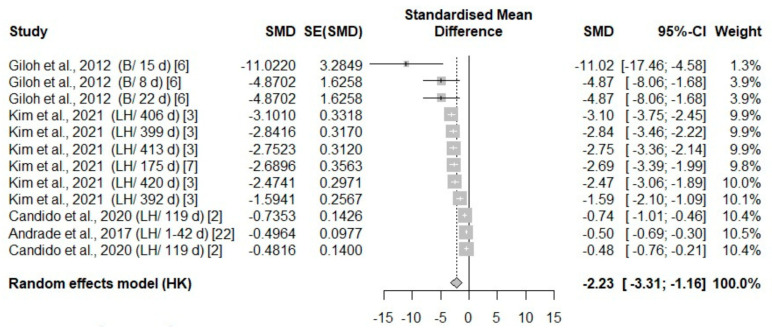
Mean difference in face surface temperature of broiler chickens (B) and laying hens (LH) of different days of age (d) under heat stress. Effect size (SMD, Standard medium difference); SE (standard deviation of SMD) [2,3,6,7,22].

**Figure 6:**
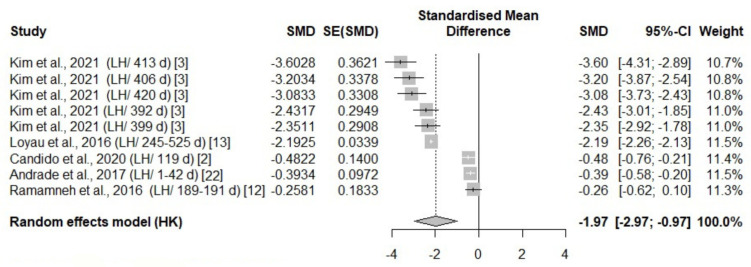
Mean difference in leg surface temperature of laying hens (LH) of different days of age (d) under heat stress. Effect size (SMD, Standard medium difference); SE (standard deviation of SMD) [2,3,12,13,22].

A total of eight studies reported thermal changes in all body areas (head parts, body, face, and leg), with an overall negative effect size (−1.85, −3.65, −2.23, −1.97, respectively) favoring exposure (Table 3). The surface temperature in all body areas undergoing heat stress was significantly higher compared to the control temperature groups in the experimental studies (Figure 3, Figure 4, Figure 5 and Figure 6).

All eligible studies that included leg surface temperature measurements were conducted in laying hens; hence, no species effect was observed. However, an age effect was identified in the leg surface temperature measurements, with a higher effect size in laying hens over 399 days of age (Figure 6). There was a significant species size effect on the other body areas (*p* < 0.05). The test for heterogeneity was significant for all parts of the body (Table 3).

#### 3.3.2. Analysis of Studies That Investigated Body Temperature Changes in Poultry During Cold Stress

**Figure 7:**
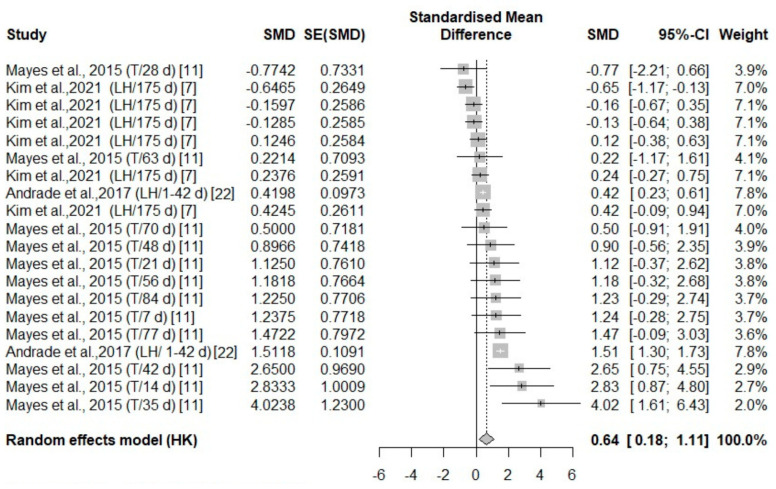
Mean difference in head parts surface temperature of turkeys (T) and laying hens (LH) of different days of age (d) under cold stress. Effect size (SMD, Standard medium difference); SE (standard deviation of SMD) [7,11,22].

**Figure 8:**
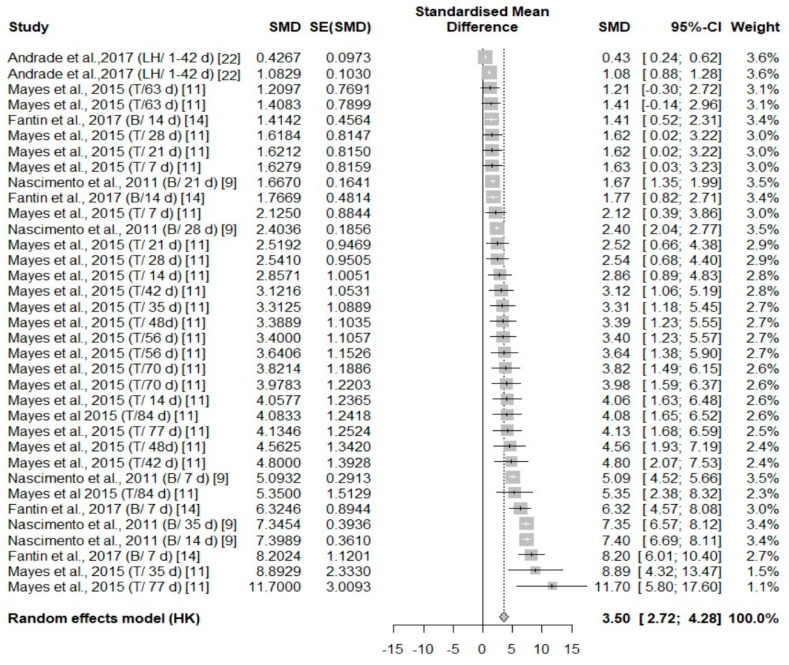
Mean difference in body surface temperature of turkeys (T), broiler (B), and laying hens (LH) of different days of age (d) under cold stress. Effect size (SMD, Standard medium difference); SE (standard deviation of SMD) [9,11,14,22].

**Figure 9:**
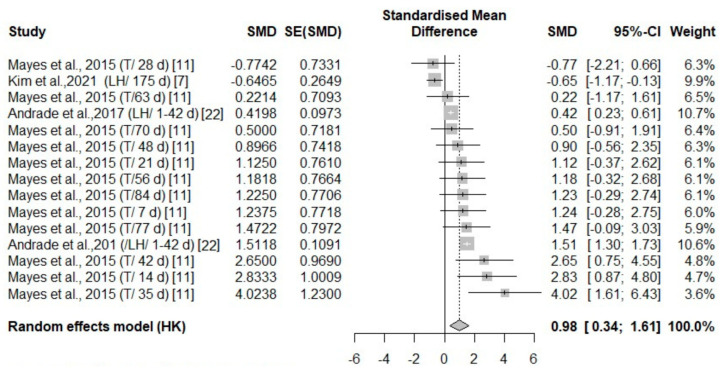
Mean difference in face surface temperature of turkeys (T) and laying hens (LH) of different days of age (d) under cold stress. Effect size (SMD, Standard medium difference); SE (standard error of SMD) [7,11,22].

**Figure 10:**
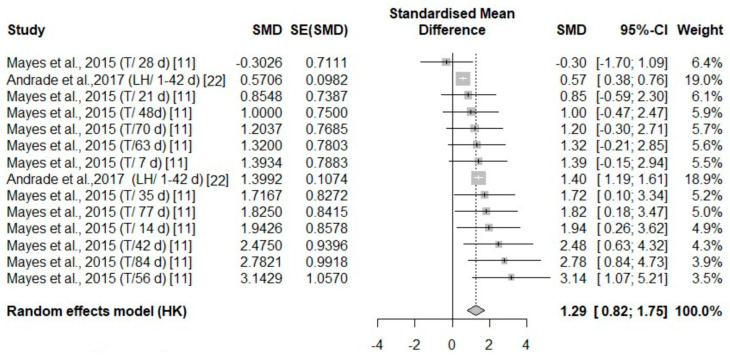
Mean difference in leg surface temperature of turkeys (T) and laying hens (LH) of different days of age (d) under cold stress. Effect size (SMD, Standard medium difference); SE (standard error of SMD) [11,22].

Overall, across five independent studies, there were thermal changes in all body areas (head, body, face, and leg) (Figure 7, Figure 8, Figure 9 and Figure 10) with a positive effect size (0.64, 3.5, 0.98, 1.29, respectively) (Table 4). The head area was the only body area to present an age and species effect secondary to cold stress exposure. The test for heterogeneity was significant for the subset of studies that included cold stress experiments (Table 4).

## 4. Discussion

A number of published review articles addressed thermal stress-related physiological responses [8,32], production performance [33], gut health [32], and egg and meat quality [15,34]. However, to our knowledge, this is the first meta-analysis that investigates the thermal changes using infrared thermography in different poultry species and body areas during cold and heat stress. One of the aims of this review was to identify the most common uses of IRT for thermal stress assessment. In the current meta-analysis, ten studies were reviewed, and ten effect sizes were generated to assess the thermal changes in different body areas of laying hens, broilers, and turkeys. Overall, the findings demonstrated that the degree of thermal surface response depends on the body area of measurement, age, treatment temperature, and species.

Surface thermal changes are cardiovascular responses that ensure thermoregulation. The physiologic responses consist of elevating the heart rate to elevate the cardiac output to increase blood circulation from the body core to the periphery areas (i.e., skin) in heat stress [3]. Conversely, peripheral vasoconstriction prevents body surface heat loss during cold stress, which is reported to be more evident in featherless areas (leg and head) [9]. The quantitative analysis revealed that feather-covered (body) and featherless areas (head, face, and leg) had a significant size effect during thermal stress, suggesting there is a thermoregulation response, regardless of the thermal stressor. In this study, the body was the feathered-covered area of interest, and it was the most common area of interest across studies. It was the only body area identified in all species and thermal stress conditions. Meanwhile, the head was the most frequently observed featherless area in the revised articles. However, the quantitative analysis did not include an assessment of turkeys undergoing heat stress.

Body surface thermal imaging was likely more commonly reported due to image-capturing limitations and anatomic differences between species. Broiler chickens have a higher body mass than laying hens and a center of gravity that moves laterally, supporting the legs, compared to laying hens’ movements in a straight line because their legs are under the center of gravity [35]. These anatomic traits make it easier to identify and track the body surface in poultry. All the components in the body area are feather-covered and had no age effect in the qualitative analysis. This is likely secondary to all birds having good feather coverage in these areas across different time points. Several studies reported feather-covered areas to have a minimal role in heat dissipation because they mainly reflect the environmental temperature [3,11,13,26]. However, the species effects found in the quantitative heat stress analysis could be associated with assessing young broilers and laying hens, and its interpretation should be taken with caution. There are reports of marked differences in feather development and growth rate between males and females and between growing broiler chickens and laying hens [36,37,38]. In contrast, the age or species of birds undergoing cold stress treatments did not affect the mean body surface temperature. This is probably secondary to initial behavioral responses to cold stress, consisting of ruffling feathers to increase isolation and crouching to reduce their body and leg surface area and heat loss [8], regardless of age or species. Furthermore, this could be an initial response, and more research is needed to understand a chronic cold stress response and the potential to produce heat depending on the amount of muscle mass (meat poultry vs. laying hen).

The head is a featherless area and presented a significant size effect in the heat and cold stress analyses, highlighting its critical role in thermoregulation, as reported in the literature [3,6,7,11,12,13]. The lack of an age effect of birds undergoing heat stress suggests the parts of the head or more generally the face are involved when the environmental temperature is above the thermoneutral zone, regardless of the age of the bird. Similar findings were reported in laying hens [22]. However, the age effect during cold stress and species-specific differences in the head parts and face surface temperatures could be associated with the variabilities between studies (I^2^ > 50%) secondary to specific experimental conditions assessed in the studies. The revised studies included several strains and species kept in different housing conditions, stocking densities, ventilation systems, thermal stress temperatures, study duration, etc. Another potential source of variability is the anatomic feature of each head part, like the shape or size of the comb, wattle, and beak. The surface and shape of some anatomic areas vary according to strains and species and are highly variable between birds [13]. This may affect the bird’s ability to exchange heat with the environment and could not be accounted for in specific anatomic body parts in this study.

Similarly, the leg had a significant effect size with no species-specific effect in the qualitative analyses. This confirms its critical role in thermoregulation as a featherless area in poultry [2,3,11,13,26]. Nääs et al. (2010) found a high correlation between environmental conditions and leg temperature due to increased blood flow in this featherless area during heat stress [39]. In addition, Loyau and colleagues (2016) suggested that heat dissipation is partly genetic, and the leg surface temperature is a reliable trait to consider in genetic selection programs. Cold stress also induces a thermoregulatory response that includes peripheral vasoconstriction to prevent body surface heat loss, which is more evident in featherless areas (leg) [9]. In this study, the leg surface temperature changes under cold stress had no age or species effect across studies, possibly due to the above-mentioned mechanism of vasoconstriction.

The verification of the quantitative analysis makes it possible to conclude that there was a high heterogeneity between studies. This variability may be caused by the different species, ages, treatments, housing conditions, and thermal imaging methodologies. Still, the general changes in the body surface temperature are consistent with the scientific literature. In future works, authors should consider the impact of thermal imaging methodologies and equipment, since some temperature variations could be caused by the equipment, not the animal or treatment [40]. To improve IRT data interpretation and implication, studies could be complemented with other animal-based variables and include egg and meat production outcomes. IRT has become more accessible during the last decades [6] and could also be paired with other technologies and algorithms (i.e., artificial intelligence). IRT images are a valuable visual input for computer vision systems and have shown potential for several welfare and clinical conditions, including behavioral changes, feather damage, and disease diagnosis [41,42].

IRT is a valuable diagnostic tool for assessing body surface temperature changes, and appropriate mitigation strategies for thermal stress should continue to be explored. The most common strategies include nutritional and environmental management, including feed restriction, nighttime feeding, use of feed additives, wet feeding, and temperature regulation with fans, foggers, or sprinkler systems [43]. However, due to increasing interest in thermal stress, other strategies like thermal conditioning at early stages and selective breeding are being studied [43]. IRT, in combination with other animal-based and resource-based indicators, has the potential to aid in animal welfare assessment in research on thermal stress mitigation in poultry.

## 5. Conclusions

IRT is a valuable tool for assessing body surface temperature changes in poultry under thermal stress. Still, more work is needed to promote its use in experimental and on-farm conditions. Head, body, and leg surface temperatures demonstrated changes under heat and cold stress. However, featherless areas provide valuable information on the stressor effect and strongly relate to the core body temperature. The mean superficial body temperature of feather-covered surfaces is a reliable variable for cold stress assessment and is likely the effect of behavioral changes to ensure insulation and control heat loss. The combined use of IRT and other welfare and physiological indicators should also be extended to other climatic conditions, and we hope it will help us understand poultry thermoregulation better and improve production practices.

## Figures and Tables

**Table 1:** Summary of studies used to assess thermal stress on body areas of poultry included in this quantitative analysis.

References	Thermal Stress	Age (Days of Age)	Species (°C)	Control Group (°C)	Groups Exposed to Heat/Cold Stress (°C)	Camera Model	Emissivity	FPA Size *	NEDT **	Measurement Uncertainty	Distance
Ramamneh et al. 2016 [12]	Heat stress	189–191	Laying hen	20.5–26.5	34.6	FLIR-T62101	0.98	320 × 240 [18]	-	+/−2 °C [18]	1 m
Kim et al. 2021 [3]	Heat stress	378–420	Laying hen	22	32	Cat S60 equipped with FLIR Lepton	-	80 × 60	-	+/−3 °C	0.8 m
Candido et al. 2020 [2]	Heat stress	1–119	Laying hen	20	35	ThermaCam b60 FLIR Systems	0.95	2048 × 1536	0.07 mK [19]	+/−2 °C	1.3 m
Kim et al. 2021 [7]	Heat/Cold stress	175	Laying hen	22	30/10	CX320; COX Co.	-	640 × 480 [20]	60 mK [20]	-	1.5 m
Layou et al. 2016 [13]	Heat stress	245–525	Laying hen	18–20	28–30	FLIR B335	Featherless-0.98 Feathered 0.896	320 × 240 [21]	50 mK [21]	+/−0.05 °C	-
Andrade et al. 2017 [22]	Heat/Cold stress	1–42	Laying hen	19–31	22–38/17–28	ThermaCAM b60 FLIR Systems	0.95	180 × 180 [19]	0.07 mK [19]	+/−2 °C	1.3 m
Giloh et al. 2012 [6]	Heat stress	1–36	Broiler chicken	21.6–30.6	35.2–38.1	PM545 FLIR System	-	320 × 240	-	+/−0.1 °C	-
Nascimento et al. 2011 [9]	Heat/Cold stress	7–35	Broiler chicken	25	32/18	TESTO 880	0.95	160 × 120 [23]	<0.1 [23]	+/−0.5 °C [23]	-
Fantin et al. 2017 [14]	Cold stress	7–14	Broiler chicken	30	24–27	Fluke TI55FT20/54/7	0.95	-	−50 mK [24]	+/−2 °C [24]	-
Mayes et al. 2015 [11]	Cold stress	7–48	Turkey	19–29	15–25	FLIR S60	1	320 × 240	-	+/−2 °C	0.91–1.22 m

* FPA: focal plane array; ** NEDT: noise equivalent differential temperature.

**Table 2:** Classification of body areas in all studies selected for this quantitative analysis.

Body Areas	Components	References
Head parts	Wattle, eye, earlobe, beak, comb	[3,6,7,11,12,13,22]
Body	Chest, back, wing, overall body	[2,3,6,9,11,12,13,14,22]
Face	General head, left side of the face, right side of the face	[2,3,6,7,11,22]
Leg	Leg, shank	[2,3,11,12,13,22]

**Table 3:** Results of thermography meta-analysis according to body area in poultry under heat stress.

Variable	Effect Size		Fixed Effect			
	Effect Size (95% Confidence Interval)	*p*-Value	Age *p*-Value	Species *p*-Value	Q Test *p*-Value	I^2^
Head parts	−1.85 [−2.56; −1.14]	<0.0001	0.45	0.02	<0.0001	99%
Body	−3.65 [−5.21; −2.08]	<0.0001	0.11	0.003	<0.0001	100%
Face	−2.23 [−3.30; −1.16]	0.0008	0.65	0.03	<0.0001	95%
Leg	−1.97 [−2.97; −0.97]	0.002	0.0005	0.20	0.0001	99%

**Table 4:** Results of thermography meta-analysis according to body area in poultry under cold stress.

Variable	Effect Size		Fixed Effect			
	Effect Size (95% Confidence Interval)	*p*-Value	Age *p*-Value	Species *p*-Value	Q Test *p*-Value	I^2^
Head parts	0.64 [0.175; 1.11]	0.01	0.004	0.03	<0.0001	87%
Body	3.50 [2.72; 4.28]	<0.0001	0.67	0.15	<0.0001	96%
Face	0.98 [0.34; 1.61]	0.005	0.05	0.24	<0.0001	87%
Leg	1.29 [0.82; 1.75]	<0.0001	0.15	0.28	<0.0001	74%

## Data Availability

Data can be made available upon request to the corresponding author.
